# Factors Associated With the Time to Next Attack in Neuromyelitis Optica: Accelerated Failure Time Models With Random Effects

**DOI:** 10.1371/journal.pone.0082325

**Published:** 2013-12-16

**Authors:** Sung-Min Kim, Junwoo Park, Sun Hee Kim, Su-Yeon Park, Jee Young Kim, Jung-Joon Sung, Kyung Seok Park, Kwang-Woo Lee

**Affiliations:** 1 Department of Neurology, Seoul National University, College of Medicine, Seoul, Korea; 2 Medical Research Collaborating Center, Seoul National University Hospital, Seoul, Korea; 3 Department of Neurology, Seoul National University, Bundang Hospital, Gyeonggi, Korea; Institute Biomedical Research August Pi Sunyer (IDIBAPS) - Hospital Clinic of Barcelona, Spain

## Abstract

**Background and Objective:**

Neuromyelitis optica (NMO) is an inflammatory demyelinating disorder of the central nervous system with a relapsing and remitting course. We aimed to identify factors associated with the time to next attack, including the effect of the natural disease course and the diverse treatment regimens, by applying a longitudinal statistical analysis to the individual attacks of each patient.

**Methods:**

In total, 184 acute attacks among 58 patients with either NMO or NMO spectrum disorder with anti-aquaporin-4 antibody were assessed retrospectively. Patient demographics, clinical characteristics at each attack, and type of treatment during inter-attack periods were assessed. The dependent variable was defined as the time from each attack to the next attack (inter-attack interval). An exponential accelerated failure time model with shared gamma frailty was adapted for statistical analysis.

**Results:**

A multivariable analysis revealed that the time from each attack to the next attack in NMO increased independently by 1.31 times (95% confidence interval (CI), 1.02–1.67; *p* = 0.035) with each additional cumulative attack experienced, by 5.34 times (95% CI, 1.57–18.13; *p* = 0.007) with combined azathioprine treatment and continued oral prednisolone, and by 4.26 times (95% CI, 1.09–16.61; *p* = 0.037) with rituximab treatment.

**Conclusion:**

The time to next attack in NMO can increase naturally in the later stages of the disease as the number of cumulative attacks increases. Nevertheless, both combined azathioprine treatment with continued oral prednisolone and rituximab treatment were also associated with a longer time to next attack, independently of the natural disease course of NMO.

## Introduction

Neuromyelitis optica (NMO) is an inflammatory demyelinating disorder of the central nervous system that involves primarily the optic nerve and the spinal cord [1,2].

As most patients with NMO experience relapsing and remitting disease courses without secondary progression [3], the rate of relapses is a major factor in their prognosis. Although several previous studies have advocated the effect of several disease-modifying treatments in NMO [4-7] based on observations of a reduced rate of relapse after the initiation of these treatments, no well-controlled studies have evaluated the compounding effects of the natural course of disease and the diverse treatment regimens on the rate of relapse in these patients.

We aimed to identify factors associated with the time to next attack, including the effect of the natural disease course and of the diverse treatment regimens, by applying a longitudinal statistical analysis[8] to the individual attacks of each patient.

## Materials and Methods

### Patients and clinical parameters

**Figure 1:**
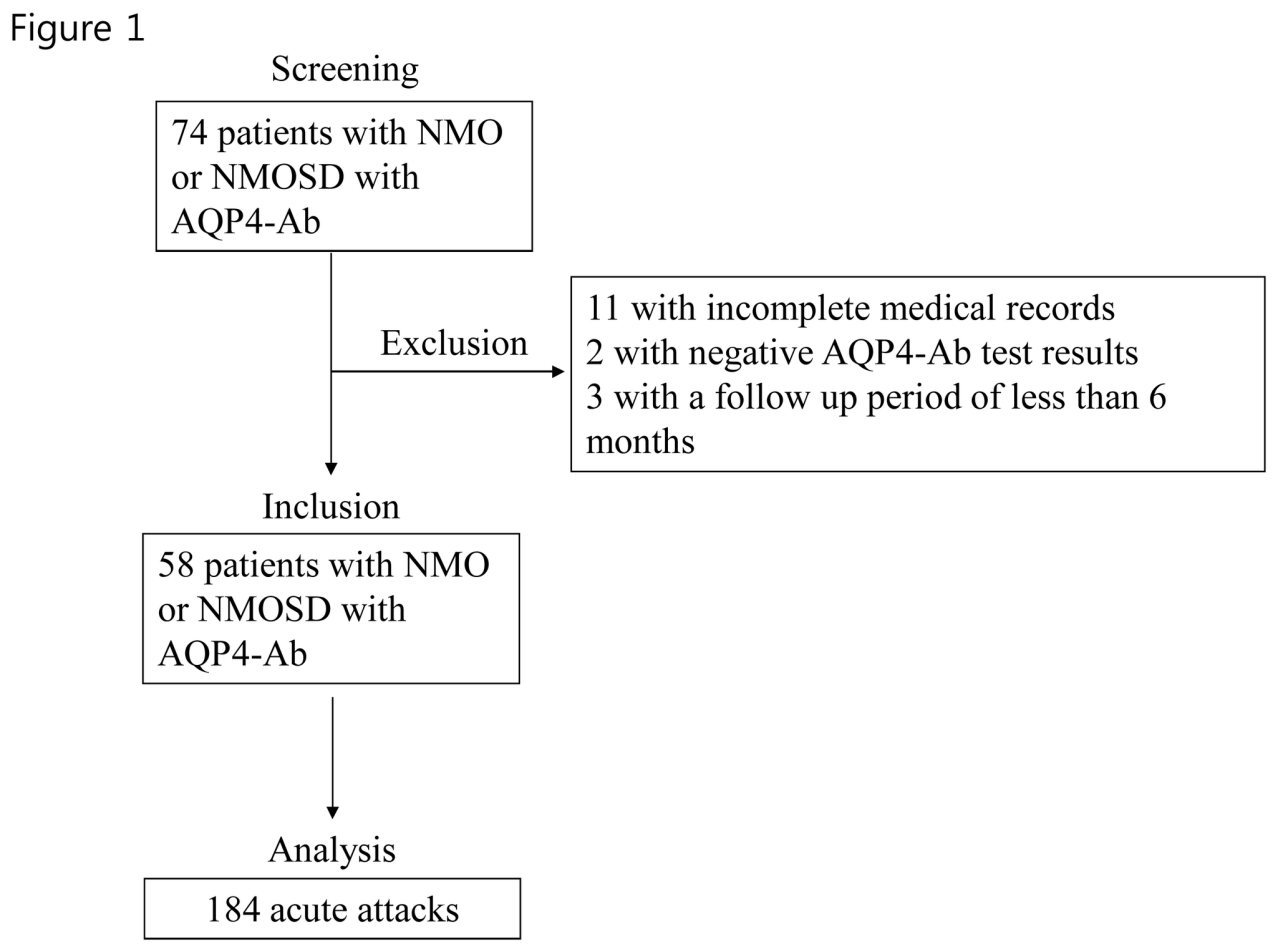
Patients screening and inclusion. In total, 184 acute attacks were identified from 58 patients with NMO or NMOSD with AQP4-Ab.

Abbreviation: AQP4-Ab, autoantibody to aquaporin-4; NMO, neuromyelitis optica; NMOSD,= neuromyelitis optica spectrum disorder.

This was a retrospective study. Seventy-four patients with either NMO or NMO spectrum disorder (NMOSD) with anti-aquaporin-4 autoantibody (AQP4-Ab) [2] who visited either the Seoul National University Hospital or the Seoul National University Bundang Hospital between September 1, 2009 and July 30, 2013 were screened. Among them, 11 patients (including 1 male; age of onset, 45.15 ± 9.82 years; all 9 patients tested were positive for AQP4-Ab) who had incomplete medical records of any of their cumulative attacks were excluded from the study. Two patients with NMO who tested negative in the AQP4-Ab assay was excluded because the disease course and/or pathomechanism of seronegative NMO might be different from those of AQP4-Ab-positive NMO [9]. Three patients who were followed for less than 6 months were also excluded. Finally, 58 patients with either NMO (n = 25) [2] or NMOSD other than NMO with AQO4-Ab (n = 33) [10] who had complete records for all of their cumulative attacks were included in our study (Figure 1). The test for AQP4-Ab was performed at the John Radcliffe Hospital, Oxford, UK, using a cell-based assay as described previously [11].

The medical records of each patient were assessed longitudinally from the time of disease onset until the time of last follow-up. An acute attack was defined as an acute episode of neurological dysfunction lasting 24 h or more and occurring more than 30 days after any previous attack [12]. In total, 184 acute attacks were identified in 58 patients. In each patient, the time from the onset of each attack to the onset of next attack (inter-attack interval) [13] was measured repeatedly [12,14].

Patient demographics, disease duration, cumulative number of attacks, and location of the attacks were assessed at the time of each recent attack and/or at the time of the first attack. The details of the treatment regimen (oral prednisolone, azathioprine, rituximab, interferon, cyclophosphamide, mitoxantrone, or methotrexate) administered during each inter-attack interval (between each recent attack and the next attack) were assessed. Among the various treatment regimens used, treatment with oral prednisolone was categorized further according to the duration of the treatment as follows: continued treatment until the next attack, treatment for more than 6 months but not until the next attack, or treatment for less than 6 months. Treatment with azathioprine was categorized according to the dosage of treatment as well as the duration of the combined prednisolone treatment, as above. Our patients who were treated with rituximab received a dose of 375 mg/m^2^ weekly, or 1000 mg separated by 2 weeks, followed by additional infusions with monitoring of CD19-positive lymphocytes [6].

### Statistical analyses

As patients with NMO can be exposed to various types of treatment during the course of their disease, a single measurement of the changes that occurred between the baseline characteristics and those recorded at follow-up visits (naive statistical analysis) would not reflect the diverse effects of those treatments [8]. Therefore, we applied a longitudinal statistical analysis to the recurrent attacks of each patient, in which the dependent variable was defined as the time from the onset of each attack to the onset of the next attack (inter-attack interval).

The activity of the disease can vary between individuals affected with NMO [15]. Moreover, it is reasonable to expect some correlation among the recurrence times of a patient. Therefore, we used a shared frailty model in which the frailty explains the heterogeneity among patients but is constant over time within a patient. We considered the frailty distribution as a gamma distribution because it fits well to failure data from a computational and analytical point of view; thus, it is the distribution that is adapted most often in a frailty model.

We adapted an accelerated failure time model (AFT), rather than the more commonly used Cox model. The AFT model can explain the relationship between the survival time and the covariates, whereas the Cox model can explain the relationship between hazard and covariates. For the AFT model with shared frailty, exponential, Weilbull, Log-logistic, and Log-normal distribution were considered for survival times: the exponential distribution was selected based on the Akaike information criterion (AIC) and the probably reasonable assumption that the risk of an NMO event occurring in a unit of time would be consistent. AIC is a measure of the goodness of fit of a model and is used to choose between competing models. Finally, we adapted an exponential accelerated failure time (AFT) model with shared gamma frailty, assuming that the unobserved patient-level factors would follow a gamma distribution [8,16-19]. A time ratio > 1 in our AFT model indicated that the variable was associated with a longer time from each attack to the next attack in NMO, whereas a time ratio < 1 indicated that the variable was associated with a shorter time to next attack [20]. The association between each variable and the time to next attack was assessed using the time ratio of the AFT model with 95% confidence intervals (CIs).

A multivariable exponential AFT model analysis was also performed using the variables that reached a *p*-value < 0.2 in the univariable analysis. In a model selection application, the optimal fitted model is identified by the minimum value of AIC. The R software version 2.15.1 with the base and survival package was used to perform all statistical analyses. All statistical tests and CIs were 2-sided with a significance level of 5%, unless specified otherwise.

### Standard protocol approval, registration, and patient consent

All patients provided their written informed consent prior to participation in the study. This study was approved by the Seoul National University Hospital Institutional Review Board (IRB number: H‑1012-023-317) and the Seoul National University Bundang Hospital (IRB number: B-1007-105-401).

## Results

The characteristics of the patients included in this study are presented in Table 1.

**Table 1:** Patient demographics and treatments.

	Number of patients (total number of attacks during follow-up)				58 (184)	
	Definite NMO/Limited NMO				25/33	
	Male/Female				7/51	
	Follow-up duration, mo^a^				70.68 ± 58.34 (7.16–348.98)	
	Mean age of onset, yr^a^				42.86 ± 14.32 (9.83–72.76)	
	Positive AQP4-Ab assay results^b^				55 (55)	
	Location of the first attack^c^			Optic nerve	24	
				Spinal cord	30	
				Brain	6	
	Location of the most recent attack^d^			Optic nerve	61	
				Spinal cord	119	
				Brain	19	
	Number of cumulative attacks^a^				3.4 ± 1.6 (1–6)	
	**Treatment during the inter-attack interval^e^**					
		Pd, duration of Tx	<6 Mo		44	
			≥6 Mo^f^		23	
			Continued Tx		35	
		AZA, dosage per day	50 mg		6	
			100 mg		18	
			150 mg		7	
		AZA and Pd, combined Tx	AZA with <6 Mo Pd		7	
			AZA with ≥ 6 Mo Pd ^f^		4	
			AZA with continued Pd		20	
		Rituximab			13	
		Interferon beta			26	
		Cyclophosphamide			5	
		Mycophenolate			6	
		Mitoxantrone			4	
		Methotrexate			3	

Abbreviations: AQP4-Ab, autoantibody to aquaporin-4; AZA, azathioprine; Mo, month; NMO, neuromyelitis optica; Pd, oral prednisolone; Tx, treatment

^a^ Expressed as mean ± standard deviation (minimum–maximum value)

^b^ Number of patients with positive results (number of patients tested)

^c^ Two patients had simultaneous involvement at 2 locations

^d^ Fifteen most recent attacks had simultaneous involvement at 2 locations

^e^ Expressed as the number of patients treated with each drug

^f^ Oral prednisolone was discontinued before the onset of the next attack

### Univariable analysis (Table 2)

**Table 2:** Univariable exponential AFT model with shared gamma frailty for the identification of predictors of time to next attack (inter-attack interval).

	**Independent variables**			**Unadjusted time ratio (95% CI)**	***p*-value**	
	Demographics					
		Sex (Ref: female)		1.21 (0.67-2.20)	0.530	
		***Disease duration***		***1.34 (1.16-1.56)***	***0.038***	
		Age at disease onset		1.01 (1.00-1.01)	0.813	
		Age at the most recent attack		1.00 (0.98-1.01)	0.666	
		***Cumulative number of attacks***		**1.34 (1.16 - 1.56)**	***0.000***	
	First attack, location					
		Optic nerve		0.82 (0.53-1.26)	0.368	
		Spinal cord		1.15 (0.75-1.76)	0.533	
		Brain		0.97 (0.5-1.91)	0.938	
	Most recent attack, location					
		***Optic nerve***		***0.56 (0.37-0.84)***	***0.005***	
		***Spinal cord***		***1.63 (1.09-2.44)***	***0.018***	
		Brain		1.00 (0.53-1.89)	0.997	
	Treatment (Ref: no treatment)					
		Pd, duration of Tx	<6 Mo	1.04 (0.65-1.66)	0.883	
			≥6 Mo ^a^	1.64 (0.91-2.98)	0.102	
			***Continued Tx***	***2.97 (1.48-5.99)***	***0.002***	
		***AZA***, ***dosage*** ^b^		***1.68 (1.19-2.37)***	***0.003***	
		AZA and Pd, combined Tx	AZA with <6 Mo Pd	0.65 (0.26-1.64)	0.366	
			***AZA with ≥6 Mo Pd*** ^a^	***7.45 (1.00-55.72)***	***0.050***	
			***AZA with continued Pd***	***6.00 (1.85-19.5)***	***0.003***	
		***Rituximab***		***6.06 (1.74-21.17)***	***0.005***	
		Interferon beta		0.64 (0.39-1.08)	0.093	
		Cyclophosphamide		2.88 (0.81-10.21)	0.102	
		Mycophenolate		1.56 (0.36-6.68)	0.549	
		Mitoxantrone		1.71 (0.45-6.54)	0.432	
		Methotrexate		1.93 (0.40-9.39)	0.415	

Abbreviations: AFT, accelerated failure time; AQP4-Ab, aquaporin-4 autoantibody; AZA, azathioprine; CI, confidence interval; F, female; M, male; Mo, month; NMO, neuromyelitis optica; other NMOSD, neuromyelitis optica spectrum disorder other than NMO; Pd, oral prednisolone; Ref, reference value; Tx, treatment

^a^ Oral prednisolone was discontinued before the next attack

^b^ Analyzed as a linear variable

The exponential AFT model revealed that a longer disease duration, a higher cumulative number of attacks, most recent attacks at the spinal cord, continued oral prednisolone treatment, higher dose of azathioprine treatment (up to 150 mg/day), azathioprine treatment combined with oral prednisolone for more than 6 months, azathioprine treatment combined with continued oral prednisolone, and rituximab treatment were significantly associated with a longer time to next attack. It also showed that most recent attacks at the optic nerve were associated with a shorter time to next attack.

### Multivariable analysis (Table 3)

**Table 3:** Multivariable exponential AFT model with shared gamma frailty for the identification of predictors of time to next attack (inter-attack interval).

	**Independent variables**		**Adjusted time ratio (95% CI)**	***p*-value**	
	***Cumulative number of attacks***		***1.31 (1.02-1.67)***	***0.035***	
	Disease duration		1.00 (0.99-1.00)	0.443	
	Age at the most recent attack		0.99 (0.97-1.01)	0.277	
	Recent attack at the optic nerve		0.64 (0.39-1.04)	0.073	
	AZA and Pd, combined Tx	AZA with <6 Mo Pd	0.73 (0.23-2.3)	0.590	
		AZA with ≥6 Mo Pd ^a^	5.70 (0.69-46.77)	0.105	
		***AZA with continued Pd***	***5.34 (1.57-18.13)***	***0.007***	
	***Rituximab***		***4.26 (1.09-16.61)***	***0.037***	
	Interferon beta		0.69 (0.35-1.35)	0.281	

Abbreviations: AFT, accelerated failure time; AZA, azathioprine; CI, confidence interval; Mo, month; Tx, treatment

^a^ Oral prednisolone was discontinued before the next attack

The cumulative number of attacks, disease duration, the most recent attacks at the optic nerve, azathioprine treatment combined with oral prednisolone, rituximab treatment, and treatment with interferon beta, cyclophosphamide, and methotrexate were included in our multivariable analysis, based on the results of the univariable analysis and the minimum value of the Akaike information criterion (AIC) [21].

Multivariable analysis revealed that the cumulative number of attacks was significantly associated with a longer time to next attack (by 1.31). Combined azathioprine treatment with continued oral prednisolone was also associated with a longer time to next attack (by 5.34). Moreover, treatment with rituximab was significantly associated with a longer time to next attack (by 4.26). Although azathioprine treatment combined with oral prednisolone for more than 6 months but discontinued before the next attack showed a tendency toward a longer time to next attack, this result did not reach statistical significance.

The duration of disease, most recent attack at the optic nerve, and treatment with interferon beta, cyclophosphamide, or methotrexate were not significantly associated with the time to next attack.

## Discussion

In this study, we demonstrated the following: 1) the time to next attack, which represents the rate of relapse inversely, can increase naturally in the later stages of the disease in NMO; and 2) both combined azathioprine treatment with continued oral prednisolone and treatment with rituximab were associated with a longer time to next attack, independently of the natural course of NMO.

Because the time to next attack in patients with NMO can be substantially influenced by the diverse range of immune-modifying and/or immune-suppressing drugs that are administered according to the severity and stage of the disease, we analyzed longitudinally the clinical characteristics of 184 attacks and the types of treatment used during the inter-attack period. Using a multivariable analysis that controlled the effect of the various treatments, we demonstrated that the time from each attack to the next attack (inter-attack interval) in NMO increased by 1.31 times with the cumulative number of attacks experienced.

We also showed that both combined azathioprine treatment with continued oral prednisolone and treatment with rituximab were independently associated with a longer time to next attack in our patients with NMO, independently of their cumulative number of attacks. This finding is important because most of the previous studies that indicated the efficacy of various immunosuppressants in NMO were based on observations of a reduced relapse rate after treatment with these drugs[6,7,22-24]. However, a reduced relapse rate after the initiation of new drugs may reflect either the suppression of disease activity by these drugs, the natural course of the disease, or the combined effects of both. Nevertheless, it seems that at least these 2 treatment regimens can actually decrease the rate of relapse in NMO, independently of the natural course of the disease, according to the results of our multivariable analysis.

The exact cause of the increased time to next attack observed in the later stage of disease in patients with NMO is not entirely clear. One of the major reasons for this finding might be that patients in the later stage of disease may not be aware of new attacks because of their pre-existing disability. However, we cannot rule out completely the possibility that some immune-modulating mechanisms, such as the recurrent exposure to the self-antigen (AQP4 in the astrocytes) after the breakdown of the blood–brain barrier [25], might play a some role in this phenomenon.

Our study had several statistical advantages. First, this study adopted the AFT model, rather than the more commonly used cox proportional hazard (PH) model [26]. The AFT model is a statistical model that is used for survival analysis and can be more advantageous than the cox PH model in that it can specify a direct relationship between survival time and independent variables, permits the estimation of the median event time, and is less affected by omitted covariables [26,27]. This was particularly important in our study because our aim was to identify factors associated with the dependent time variable, and to estimate their effects on the time to relapse. Second, we used variables obtained from repeated observations of individual attacks (longitudinal analytical techniques), rather than from only one observation for each patient (naïve techniques, per se logistic regression) [8]; the latter method has been used in several previous studies of NMO [28-31]. However, a patient with NMO is typically exposed to a diverse range of immune-modifying and/or immune-suppressing drugs that change according to the severity and stage of the disease. Therefore, controlling for the effects of these diverse treatment regimens in the disease course is necessary when evaluating the factors that are independently associated with the time to next attacks in NMO. This can only be achieved by analyzing each repeated attack in individual patients using a longitudinal analytical technique [8], as described here. Third, the shared frailty model can reflect effects that occur within each of the patients (random effects), which is necessary because the disease course and prognosis in NMO can vary considerably between affected individuals [15].

Despite the important findings described above, our study also had limitations. First, this was a retrospective investigation, and the number of attacks included in our study (184 attacks) was relatively small for multivariable analysis. Second, some clinical parameters, such as the opticospinal intervals or results of cerebrospinal fluid studies, were not included in our analyses. As those variables could be obtained only for some of our patients (per se; length of the spinal cord lesions cannot be obtained when the most recent attack occurs in the optic nerve or brain), including these variables in our multivariable analysis would eventually have decreased the statistical power of our analysis by reducing the sample size.

In summary, we found that the time to next attack in NMO was independently associated with the cumulative number of attacks, combined azathioprine treatment with continued oral prednisolone, and treatment with rituximab. Our findings are important for the following reasons: 1) we demonstrated natural changes in disease activity after controlling for the effect of various treatment regimens; 2) although numerous previous studies on NMO reported a reduced rate of relapse after treatment with the various immunosuppressant drugs [6,7,22-24], this type of analytic approach may be substantially affected by the possible natural change in the disease activity over time. Our study showed for the first time that some immunosuppressant drugs can be effective in preventing the relapse of NMO, independently of the natural change in disease activity; and 3) this study evaluated the usefulness and the pitfalls of a new clinical outcome, i.e., time to next attack [32], in assessing the effect of treatment in NMO.
